# Sphenoid sinus mucosal thickening in the acute phase of pituitary apoplexy

**DOI:** 10.1007/s11102-017-0804-z

**Published:** 2017-04-18

**Authors:** Mueez Waqar, Robert McCreary, Tara Kearney, Konstantina Karabatsou, Kanna K. Gnanalingham

**Affiliations:** 1 Department of Neurosurgery, Greater Manchester Neuroscience Centre, Salford Royal Foundation Trust (SRFT), Stott Lane, Salford, M6 8HD UK; 2 Department of Neuroradiology, Salford Royal NHS Foundation Trust, Manchester, UK; 3 Department of Endocrinology, Salford Royal NHS Foundation Trust, Manchester, UK; 4 Manchester Academic Health Sciences Centre, University of Manchester, Manchester, UK

**Keywords:** Pituitary apoplexy, Sphenoid sinus, Sphenoid sinus mucosa, Mucosal thickening, Magnetic resonance imaging

## Abstract

**Purpose:**

In pituitary apoplexy (PA), there are preliminary reports on the appearance of sphenoid sinus mucosal thickening (SSMT). SSMT is otherwise uncommon with an incidence of up to 7% in asymptomatic individuals. The aim of this study was to evaluate the incidence and clinical significance of SSMT in patients with PA and a control group of surgically treated non-functioning pituitary adenomas (NFPAs).

**Methods:**

Retrospective review of clinical and imaging variables in PA and NFPA patients. Sphenoid sinus mucosal thickness was measured on the presenting MRI scan by a blinded neuroradiologist. Pathological SSMT was defined as >1 mm adjacent to the pituitary fossa. Forward stepwise logistic regression was used to identify factors associated with SSMT.

**Results:**

There were 50 NFPA and 47 PA patients. PA patients were managed conservatively (N = 11) or surgically (N = 36). The median sphenoid sinus mucosal thickness was greater in the PA than NFPA groups (2.0 vs. 0.5 mm; p < 0.001). In multivariate analysis of both the PA and NFPA groups, the presence of PA was the only factor associated with SSMT (OR 0.043, 95% CI 0.012–0.16; p < 0.001). In multivariate analysis of the PA group alone, a shorter time from symptom onset to presenting MRI scan (OR 0.12, 95% CI 0.026–0.54; p = 0.006) and a more severe grade of apoplexy (OR 7.29, 95% CI 1.10–48.40; p = 0.04), were associated with SSMT.

**Conclusion:**

The incidence of SSMT is higher in patients with PA, especially during the acute phase of PA. The aetiology of SSMT in PA is unclear and may reflect inflammatory and/or infective changes.

## Introduction

Classical pituitary apoplexy (PA) is a clinical syndrome of acute onset severe headache associated with nausea, meningism, visual impairment (reduced visual acuity and ophthalmoplegia) and decreased level of consciousness [1, 2]. Electrolyte imbalance and raised inflammatory markers are relatively common and if not adequately managed PA carries significant morbidity and mortality [1, 2]. PA is secondary to haemorrhage and/or infarction into a pituitary adenoma, with a reported incidence of about 2–7% among patients with pituitary adenomas [1, 2]. Precipitating factors include hypertension, anticoagulant therapy, coagulopathy, major surgery, dopamine agonist therapy, pregnancy and radiation therapy [2]. Surgical intervention is frequently necessary in these patients, although increasingly in carefully selected patients, medical management may be appropriate [3].

The sphenoid sinus is the gateway to the pituitary fossa during trans-sphenoidal surgery. Sphenoid sinus disease can manifest radiologically as mucosal thickening and/or fluid collection, leading to sphenoid sinus opacification on imaging [4]. However, the sphenoid sinus is the least likely of the paranasal sinuses to be affected in healthy individuals [5, 6]. Indeed, the reported prevalence of incidental sphenoid sinus mucosal thickening (SSMT) varies between 2–7% [5, 6]. In a Norwegian study of MRI scans of 982 individuals aged 50–65 recruited into epidemiological trials, SSMT was observed in only 136 cases with 1963 sinuses (one aplastic sphenoid sinus), representing a rate of 7% [6].

There is evidence to suggest SSMT may be seen on the preoperative MRI scans of patients with PA, although the reported incidence varies and existing study sample sizes are relatively small. In one study, 7/11 (64%) patients had evidence of SSMT in the acute phase of PA [4]. Other studies have reported higher rates of 22/28 (79%) [7] and 17/19 (89%) [8]. One study also reported that the presence of SSMT may be correlated with a poorer clinical status at presentation of PA [7].

Thus the incidence and clinical significance of SSMT in PA is relatively unclear at present. Studies to date have been small in size and lacked multivariate statistics to identify factors associated with SSMT. The aim of this single-centre study was to evaluate the incidence and clinical significance of SSMT in patients with PA, using a larger sample size and multivariate analysis.

## Methods

### Patient selection

This single-centre study retrospectively identified patients entered prospectively into an electronic database of pituitary cases treated by two surgeons between 2007 and 2016. The following patients were included

1. *Classical pituitary apoplexy (PA) group:* As defined by the Society of Endocrinology, refers to a clinical syndrome, characterised by sudden onset of headache, vomiting, visual impairment and decreased consciousness caused by haemorrhage and/or infarction of the pituitary gland [2]. Haemorrhage and/or infarction were confirmed using at least one or more of the following: imaging (T1/2 weighted magnetic resonance imaging), histopathology or intra-operative findings (i.e. evidence of pre-existing haemorrhage within the pituitary adenoma).
2. *NPFA group:* Consecutive series of surgically treated patients with non-functioning pituitary adenomas with no clinical evidence of pituitary apoplexy pre-operatively, as defined above.

### Data collection

Data concerning clinical presentation, demographics, tumour histology, treatment and follow-up were collected from electronic patient records.

For the PA group, the Pituitary Apoplexy Score (PAS) was calculated based on documentation of clinical assessment at presentation as previously described [2]. The cumulative PAS ranges from 0 to 10 and gives weight to defects in visual acuity (0–2), visual fields (0–2), eye movements (0–2) and conscious level as assessed by the patient’s Glasgow Coma Scale (GCS; 0–4). A PAS of ≥4 may be considered significant [9]. The clinical severity of apoplexy grade was also calculated as described by Liu et al. (outlined in Table 1) [2, 7].

**Table 1:** Clinical features of the PA group

	Conservatively managed (N = 11)	Surgically managed (N = 36)
Presenting feature		
Headache	10 (91%)	32 (89%)
Nausea/vomiting	5 (45%)	20 (56%)
Visual field defect	5 (45%)	21 (58%)
Visual acuity defect	3 (27%)	15 (42%)
Ocular paresis	3 (27%)	16 (44%)
Reduced GCS	1 (9%)	3 (8%)
Evidence of haemorrhage or infarction		
Radiological	11 (100%)	28 (78%)
Intra-operative	–	34/34 (100%)^a^
Histopathology	–	27 (75%)
PAS (reference 2)		
<4	10 (91%)	27 (75%)
≥4	1 (9%)	9 (25%)
Mean ± SD (range)	1.5 ± 1.4 (0–4)	2.3 ± 1.6 (0–6)
Severity of apoplexy grade^b^ (reference 7)		
Grade 1: symptoms without neurological deficits	3/11 (27%)	4/35 (11%)
Grade 2: symptoms + cranial nerve deficits	7/11 (64%)	29/35 (83%)
Grade 3: symptoms + cranial nerve deficits + reduced GCS	1/11 (9%)	2/35 (6%)

^a^In two cases, the operative notes did not describe the intraoperative pituitary appearance in sufficient detail

^b^One patient managed surgically was not classifiable according to the severity of apoplexy grading system due to a presentation with headaches and reduced GCS, but without evidence of cranial nerve palsy

Tumour histology was categorised into 3 groups based on the cell type found on histopathological assessment: null-cell, FSH/LH and other. The other group contained tumours that were biologically inactive, but demonstrated positive immunostaining for ACTH, TSH and/or prolactin.

### Imaging review

**Fig. 1:**
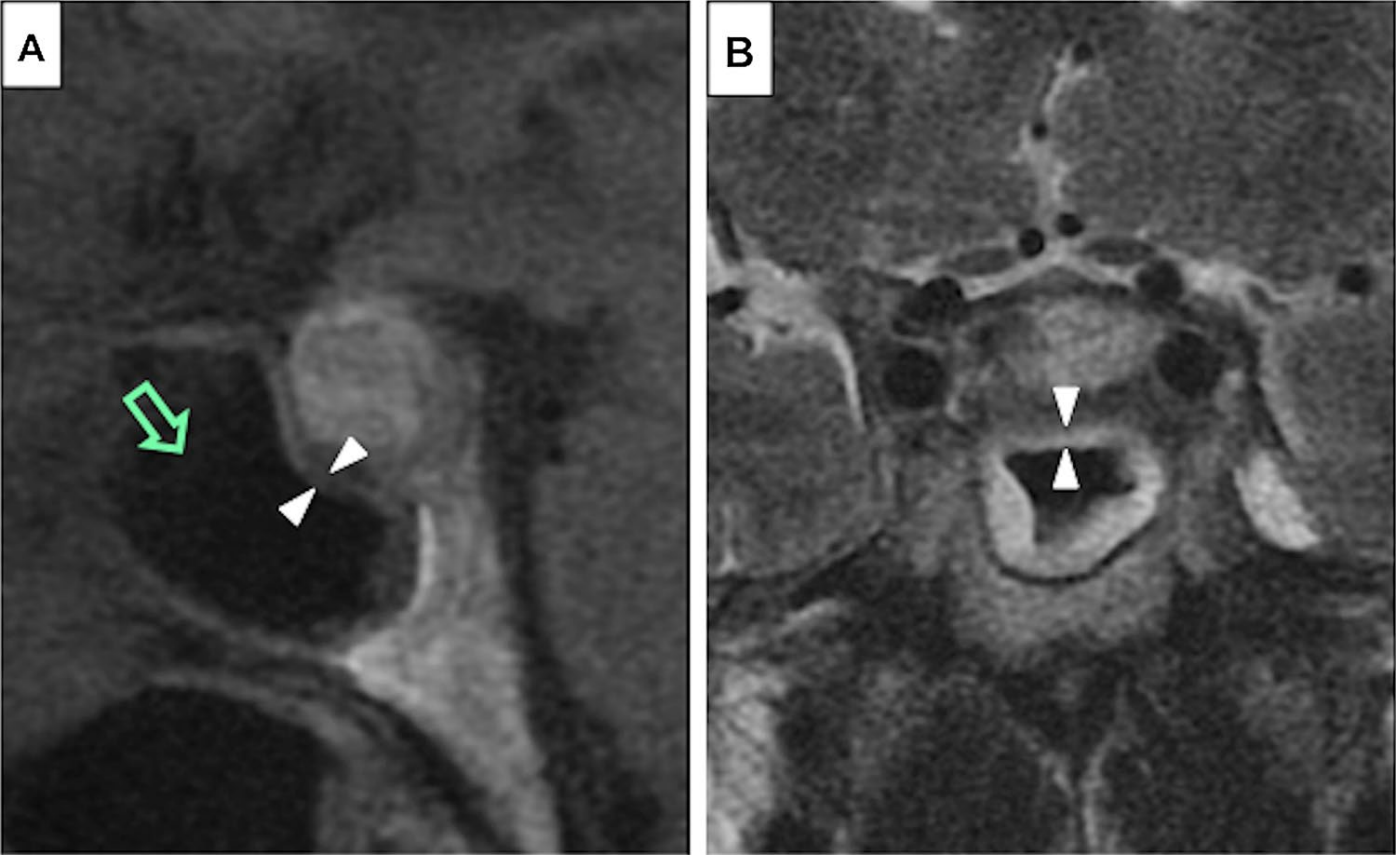
Sphenoid sinus mucosal thickness measurements were made as indicated by the white arrowheads on T1-weighted sagittal (**a**), or T2-weighted coronal MRI sequences (**b**). Sphenoid sinus opacification was assessed as the level of aeration of the sphenoid sinus (*green open arrow* in (**a**))

Sphenoid sinus measurements were performed independently by a neuroradiologist blinded to clinical data and radiology reports. Sphenoid sinus mucosal thickness was measured on the adjacent sinus wall to the pituitary to the nearest 0.5 mm on sagittal T1-weighted and/or coronal T2 weighted MRI sequences (Fig. 1). Contrasted images were used when available. A mucosal thickness of up to 1 mm was considered normal and the mucosal thickness was categorised into 3 categories (<1, 1–3 and >3 mm), as described previously [4]. The degree of sinus opacification (0–25, 25–50, 50–75, 75–100%) and the sinus type (i.e. sellar, pre-sellar and chonchal) were also recorded [10].

Pituitary tumour volumes were derived by the ellipsoid method using the three largest radii in the sagittal (a), coronal (b) and axial planes (c), as previously described (4/3**a.b.c) [11]. The time to scan was defined as the time from symptom onset where available or hospital admission, to the time of the first MRI head scan.

### Statistics

Statistical analysis was performed using SPSS version 22 (SPSS Inc., Chicago, Illinois, USA). Means for normally distributed data were compared using independent *t* tests. Categorical variables were compared using tests of proportions (Fisher’s Exact, Chi-squared). Values were rounded to whole numbers or given to two decimal places, as appropriate.

Multivariate analysis was performed to identify factors associated with SSMT using a forward step-wise logistic regression model. Variables were included if at the univariate level there was association with SSMT to a statistical significance of p < 0.1. SSMT was categorised as abnormal if >1 mm.

## Results

### Demographics

Forty seven patients with PA and 50 surgically treated NFPAs were included. The mean age at presentation was 54 ± 15 years (range 23–88 years) for the PA group versus 61 ± 14 years (range 27–88 years) for the NFPA group. The PA group was significantly younger (independent *t* test, t = 2.25, p = 0.03). There was a male predominance in both the PA and NFPA groups, with a male:female ratio of 33:14 and 32:18, respectively. There was no significant difference in gender distribution between the groups (Fisher’s Exact, p = 0.53).

### Clinical features of PA group

Clinical features of the PA group are shown in Table 1. Precipitating factors were identified in 15 cases:11 cases of existing hypertension and 4 cases of antiplatelet/anticoagulant use. Headache was the commonest presenting symptom in 42/47 cases (89%).

The mean PAS was similar between the conservatively and surgically managed groups (independent *t* test, p = 0.14; Table 1). The proportion of these groups with a PAS ≥ 4 was also similar between the conservatively and surgically managed groups (9.1 vs. 25.0%, Fisher’s Exact, p = 0.41; Table 1). The severity of apoplexy grade was similarly distributed between the groups (Chi-squared = 1.92, p = 0.38; Table 1).

### Management of PA group

Most patients with PA were treated with trans-sphenoidal surgery (36/47, 77%) and a smaller proportion were managed non surgically (11/47, 23%). Two patients in the conservatively managed group of PA patients eventually had surgery at 10 and 23 months for significant residual adenoma that was non-functioning in one patient and growth hormone secreting in another. Given that the presentation with apoplexy was managed conservatively, these patients remained in the conservatively managed group. In the surgical group, the median time to surgery after presentation was 5 days (range 1–167 days).

Five (14%) patients with PA had postoperative complications, including 1 case each of post-op CSF leak (managed with a lumbar drain), hyponatraemia, depression, hospital acquired pneumonia and atrial fibrillation. Three patients in the surgically managed PA group underwent further surgery for residual tumours and 8 received radiotherapy.

The majority (45/47, 96%) of patients with PA required hormone replacement in the form of: steroids (38/47, 81%), thyroxine (38/47, 81%), sex hormones (28/47, 60%), growth hormone (25/47, 53%) and desmopressin (3/47, 6%) at their last clinical review.

### Imaging characteristics

The median time to first MRI scan was 7 days for the PA group (range 1–224 days). This time interval was greater than 4 weeks in 5 patients who presented in a delayed fashion to the local endocrine team. Two out of the 5 patients also had complex medical/biochemical disorders that required management prior to the first MRI scan. Once admitted to the neurosurgical centre, the median time to MRI scan was 1 day (range 1–19 days).

Imaging characteristics are shown in Table 2. The majority of PA and NFPA patients had a sellar variant of sphenoid sinus, with no significant difference in the distribution of sinus types between the groups (Chi-squared = 1.73, p = 0.42; Table 2). A greater degree of sinus opacification was present in patients with PA (Chi-squared = 22, p < 0.001; Table 2).

**Table 2:** Imaging characteristics of patients with PA (N = 47) and NFPA (N = 50)

	PA (n = 47)	NFPA (n = 50)	p
Sphenoid sinus			
Type			
Sellar	40 (85%)	46 (92%)	0.42
Presellar	6 (13%)	4 (8%)	
Conchal	1 (2%)	0 (0%)	
Opacification (%)			
0–25	31 (66%)	50 (100%)	<0.001
25–50	8 (17%)	0 (0%)	
50–75	2 (4%)	0 (0%)	
75–100	6 (13%)	0 (0%)	
Mucosal thickness (mm)			
≤1	18 (38%)	47 (94%)	<0.001
1–3	18 (38%)	3 (6%)	
>3	11 (23%)	0 (0%)	
Median (range)	2.0 (0.5–6.0)	0.5 (0.5–2.0)	
Tumour volume (cm^3^)			
Median (range)	3.7 (0.7–26.5)	4.6 (1.1–25.6)	0.67

The median (range) sphenoid sinus mucosal thickness was 2.0 mm (0.5–6.0 mm) in the PA and 0.5 mm (0.5–2.0 mm) in NFPA groups, respectively (Mann–Whitney, p < 0.001). Sphenoid sinus mucosal thickness greater than 1 mm was noted in a higher proportion of PA (29/47; 62%) than NFPA group (3/50; 6%) (Chi square = 35, p < 0.001; Table 2). Sphenoid sinus mucosal thickness greater than 3 mm was only apparent in the PA, and not NFPA, group (23 vs. 0%; Table 2). The median (range) sphenoid sinus mucosal thickness was similar between the conservative −2.0 mm (0.5–4.0 mm), and surgically managed −2.0 mm (0.5–6.0 mm), PA patients (Mann–Whitney, p = 0.53).

**Fig. 2:**
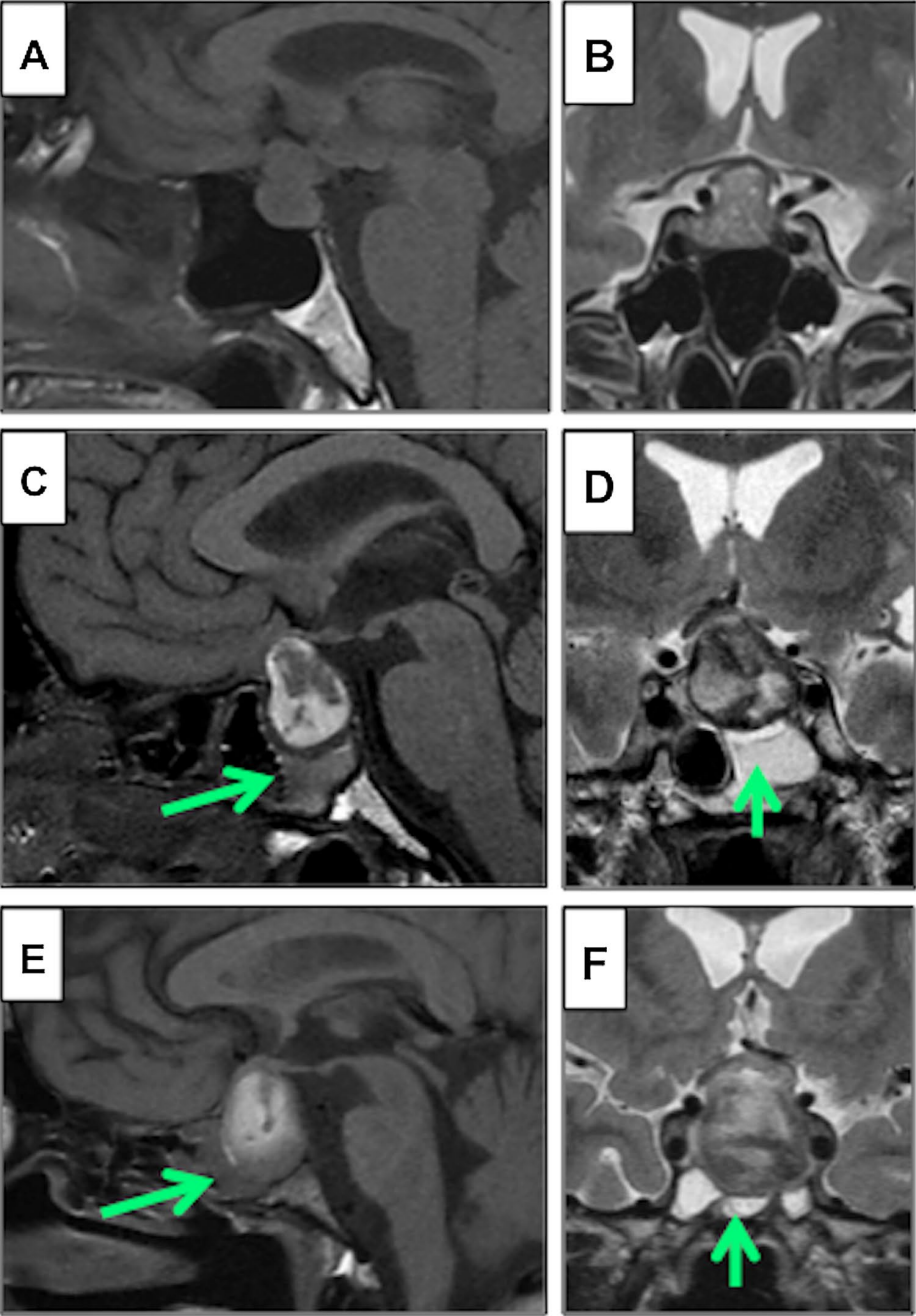
Examples of sphenoid sinus disease as measured by the sphenoid sinus mucosal thickness on sagittal T1-weighted (**a, c, e**) and coronal T2-weighted (**b, d, f**) MRI sequences. **a, b** A 66 year old male with an incidental NFPA with chiasmal compression, but no significant SSMT preoperatively. **c, d** A 56 year old male presented with acute onset headaches, nausea, bilateral field defects and reduced visual acuity. MRI showed increased signal on T1-weighted imaged consistent with haemorrhage/infarction of a pituitary adenoma and there was significant SSMT (2.5 mm) with obliteration of the left side (25–50%: *green arrow*). **e, f** A 24 year old male presented acutely with confusion and refractory hyponatraemia. Imaging revealed high signal on T1-weighted MRI and there was marked SSMT (5 mm), with near complete obliteration of the sinus space bilaterally (75–100%: *green arrow*)

**Fig. 3:**
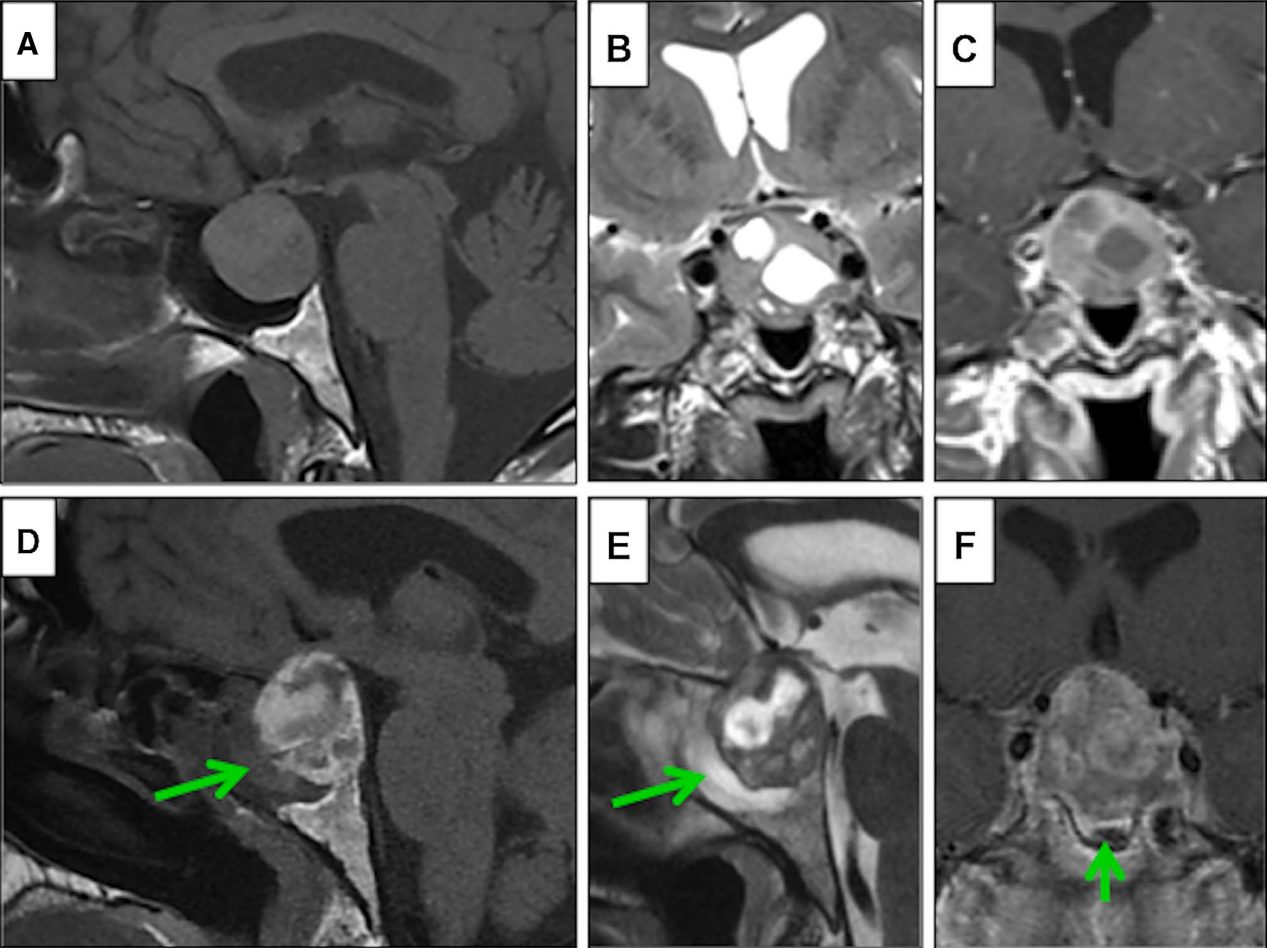
MRI changes in a patient presenting pre and post-apoplexy. This 53 year old male presented with generalised joint aches and secondary hypothyroidism was diagnosed biochemically. MRI revealed a pituitary adenoma with mild indentation of optic chiasm (**a** sagittal T1-weighted; **b** coronal T2-weighted MRI; **c** coronal T1 MRI with gadolinium enhancement). There was no evidence of significant sphenoid sinus disease. Just before admission for surgery, he developed sudden onset frontal headaches, nausea and a left 6th nerve palsy. Repeat MRI within 24 h of presentation, revealed an increase in size of the pituitary lesion with likely tumour bleed and/or infarction (**d** sagittal T1-weighted MRI; **e** sagittal T2-weighted MRI; **f** coronal T1 MRI with gadolinium enhancement). The MRI also revealed marked SSMT and near complete obliteration of the sphenoid sinus (*arrows*). During emergency trans-sphenoidal surgery, the sphenoid sinus mucosa was noted to be very abnormal and inflamed and there was evidence of acute haemorrhage and infarction within the pituitary adenoma, as also confirmed on subsequent histology. His visual fields and eye movements were normal at 6 months post-op

Case examples of SSMT in NFPA and PA patients are shown in Fig. 2. Figure 3 demonstrates a case where imaging was available pre and post-apoplexy, demonstrating the development of SSMT shortly after the symptoms of PA became apparent.

Multivariate analysis of both PA and NFPA cases revealed PA to be the only factor associated with SSMT (OR 0.043, 95% confidence interval = 0.012–0.16; p < 0.001) (Table 3).

**Table 3:** Multivariate analysis to derive factors associated with SSMT (all patients included; N = 97)

Variable	Subcategory	Incidence of SSMT (%)	Univariate analysis		Multivariate analysis^a^	
			Test	p	Odds ratio (95% CI)	p
Mean age	≤57	19/44 (43%)	Fisher’s exact	0.08	0.72 (0.24–2.12)	0.55
	>57	13/53 (25%)				
Gender	Male	23/65 (35%)	Fisher’s exact	0.50	Not included	–
	Female	9/32 (28%)				
Group	Apoplexy	29/47 (62%)	Fisher’s exact	<0.001	0.043 (0.012–0.16)	<**0.001**
	NFPA	3/50 (6%)				
Histological origin	Null cell	15/31 (48%)	Chi-squared (Chi = 5.60)	0.06	0.83 (0.44–1.57)	0.56
	LH/FSH	9/41 (22%)				
	Other	8/25 (32%)				
Sphenoid sinus type	Sellar	26/86 (30%)	Chi-squared (Chi = 4.09)	0.13	Not included	–
	Pre-sellar	6/10 (60%)				
	Conchal	0/1 (0%)				
Median tumour volume (cm^3^)^b^	≤3.8	15/49 (31%)	Fisher’s exact	0.67	Not included	–
	>3.8	17/48 (35%)				

The dependant variable of sphenoid sinus mucosal thickness greater than 1 mm was considered abnormal

^a^Variables were entered into a logistic regression model where p < 0.10

^b^Categorised as less than versus more than median value (3.8 for all 97 patients)

Multivariate analysis of the PA group alone, revealed that the time to scan (OR 0.12, 95% confidence interval = 0.026–0.54; p = 0.006) and severity of apoplexy grade (OR 7.29, 95% confidence interval = 1.10–48.40; p = 0.04), were the most important factors associated with SSMT (Table 4).

**Table 4:** Multivariate analysis look at factors associated with SSMT (PA group only; N = 47)

Variable	Subcategory	Incidence of SSMT (%)	Univariate analysis		Multivariate analysis^a^	
			Test	p	Odds ratio (95% CI)	p
Mean age	≤54	15/23 (65%)	Fisher’s exact	0.77	Not included	–
	>54	14/24 (58%)				
Gender	Male	23/33 (70%)	Fisher’s exact	0.11	Not included	–
	Female	6/14 (43%)				
PAS (reference 2)	<4	22/37 (60%)	Fisher’s exact	0.72	Not included	–
	≥4	7/10 (70%)				
Severity of apoplexy grade (reference 7)	1	1/7 (14%)	Chi-squared (Chi = 7.53)	0.02	7.29 (1.10–48.40)	**0.04**
	2	25/36 (69%)				
	3	2/3 (67%)				
Time to scan	≤1 week	20/24 (83%)	Fisher’s exact	0.003	0.12 (0.026–0.54)	**0.006**
	>1 week	9/23 (39%)				
Histological origin	Null cell	14/23 (61%)	Chi-squared (Chi = 1.44)	0.49	Not included	–
	LH/FSH	7/9 (78%)				
	Other	8/15 (53%)				
Sphenoid sinus type	Sellar	23/40 (58%)	Chi-squared (Chi = 5.63)	0.06	1.14 (0.23–5.76)	0.88
	Pre-sellar	6/6 (100%)				
	Conchal	0/1 (0%)				
Median tumour volume (cm^3^)^b^	≤3.7	13/24 (54%)	Fisher’s exact	0.37	Not included	–
	>3.7	16/23 (70%)				

The dependant variable of sphenoid sinus mucosal thickness greater than 1 mm was considered abnormal

^a^Variables were entered into a logistic regression model where p < 0.10

^b^Categorised as less than versus more than median value (3.7 for PA group)

## Discussion

The main finding of this study was the strong association between PA and SSMT. Almost two-thirds of patients with PA had evidence of SSMT on the MRI scan at presentation, compared to 6% of NFPAs. Additionally, a shorter time interval between symptom onset and the initial MRI scan emerged as the only reliable predictor of SSMT (i.e. 83 vs. 39% of patients when duration was longer than 1 week). An increasing severity of apoplexy, graded using the system described by Liu et al. [7], also emerged as a significant predictor of SSMT, though the confidence interval was large. Our study is the first to report these findings using multivariate analysis.

The incidence of SSMT in PA varies in the literature. Arita et al. were one of the first to describe it in 9/11 (82%) patients with acute PA versus a rate of 15% in a group of NFPAs [4]. Liu et al. expanded on this work and reported an incidence of 22/28 (79%) patients [7]. Semple et al. also reported definite or subtle thickening in 17/19 patients with PA (89%) [8]. Lower rates have been reported in the paediatric literature, with SSMT in only 2 out of 9 (22%) adolescents aged 14–21 [12]. Overall we found a lower rate (62%) than reported, though our study included a larger cohort of patents presenting with PA.

There are several potential reasons for differences in the reported incidence of SSMT. The first relates to the methodology employed to measure SSMT. SSMT can be difficult to assess reliably [6]. In several studies, detailed descriptions of how the SSMT was measured in the setting of PA, are lacking (e.g. MRI sequences used and plane of measurement) [4, 7, 8]. In the present study, the use of a standardised methodology and a blinded neuroradiologist to review imaging, were used to minimise potential observer bias.

Secondly, as observed in the present study, time to scan is a key variable and existing studies have not always described this variable in detail. Arita et al. described 3 patients with minimal or no SSMT in whom the first MRI scan was obtained in the chronic phase, a finding consistent with the our observations [4].

Thirdly, differences in the severity of PA may have influenced the rate of SSMT. Liu et al. reported SSMT in 16/16 (100%) versus 6/12 (50%) patients with and without neurological deficits, respectively [7]. Our findings were comparable and SSMT was present in 27/39 (69%) versus 1/7 (14%) patients with and without neurological deficits, respectively. Furthermore, almost a quarter (11/47, 23%) of patients in our study had milder, conservatively managed PA. Studies that included only surgically management PA patients have reported a higher rate of SSMT. Indeed, the highest incidence of SSMT in the literature has been reported by Semple et al., who included only surgically managed PA patients [8]. Interestingly, the severity of apoplexy as assessed by the PAS was not associated with SSMT in this study. This may be related to the fact that PAS is heavily influenced by visual findings.

The aetiology of SSMT in the context of PA remains unclear. SSMT is usually an indicator of sphenoid sinusitis, which can be inflammatory or infective in aetiology [13]. At present, there is little or no evidence to support either hypothesis. Agrawal et al. presented two cases of patients presenting with PA, who had biopsies of the sphenoid sinus mucosa [14]. They found a moderate degree of inflammation in both cases. They compared these findings to two patients with electively resected pituitary adenomas, without evidence of SSMT radiologically. They reported that these patients also had evidence of a moderate grade of inflammation of the sphenoid sinus mucosa [14]. Although it is difficult to draw conclusions from this limited analysis, it does suggest that SSMT may not be just a marker of inflammation. An alternative hypothesis is that SSMT reflects an infective process that may be related in some way to the development of PA. In support of this hypothesis, a case of cavernous sinus thrombosis secondary to sphenoid sinusitis has been reported [15]. We are currently comparing the microbiological flora of patients presenting with PA to other pituitary tumours. The spread of inflammation or infection may be facilitated by erosion of the sellar dura and bony floor, frequently apparent in cases of PA.

Limitations of this study includes its retrospective design. However, apoplexy is a relatively rare condition making prospective studies difficult. Our study is the largest to explore the link between SSMT and PA. We also did not undertake a more detailed histological or microbiological study of the sphenoid sinus mucosa. There was also a lack of serial imaging analysis to explore the temporal nature of the sphenoid sinus mucosal changes in greater detail. These were beyond the scope of the present study.

## Conclusion

In this retrospective single-centre study, we observed an association between SSMT and PA, especially in the acute phase of PA. SSMT had a higher incidence in patients with neurological deficits, acting as marker of a more severe grade of PA. The aetiology of SSMT in PA is unclear and could represent either primary infective sphenoid sinusitis that potentially contributed to the development of PA, and/or spread of a neighbouring inflammatory process in the infarcted or haemorrhagic pituitary adenoma. Future studies are required to investigate this further.
